# Classification and distribution of freshwater microplastics along the Italian Po river by hyperspectral imaging

**DOI:** 10.1007/s11356-022-18501-x

**Published:** 2022-02-23

**Authors:** Ludovica Fiore, Silvia Serranti, Cristina Mazziotti, Elena Riccardi, Margherita Benzi, Giuseppe Bonifazi

**Affiliations:** 1 Department of Chemical Engineering, Materials & Environment, Sapienza University of Rome, Via Eudossiana 18, 00184 Rome, Italy; 2 ARPAE, Regional Agency for Environmental Prevention and Energy of Emilia-Romagna, Oceanographic Unit Daphne - V. le Vespucci 2, 47042 Cesenatico, FC Italy

**Keywords:** Freshwater microplastics, Po river, Environmental pollution, Plastic litter, Hyperspectral imaging, Hierarchical classification

## Abstract

In this work, freshwater microplastic samples collected from four different stations along the Italian Po river were characterized in terms of abundance, distribution, category, morphological and morphometrical features, and polymer type. The correlation between microplastic category and polymer type was also evaluated. Polymer identification was carried out developing and implementing a new and effective hierarchical classification logic applied to hyperspectral images acquired in the short-wave infrared range (SWIR: 1000–2500 nm). Results showed that concentration of microplastics ranged from 1.89 to 8.22 particles/m^3^, the most abundant category was fragment, followed by foam, granule, pellet, and filament and the most diffused polymers were expanded polystyrene followed by polyethylene, polypropylene, polystyrene, polyamide, polyethylene terephthalate and polyvinyl chloride, with some differences in polymer distribution among stations. The application of hyperspectral imaging (HSI) as a rapid and non-destructive method to classify freshwater microplastics for environmental monitoring represents a completely innovative approach in this field.

**Supplementary Information:**

The online version contains supplementary material available at 10.1007/s11356-022-18501-x.

## Introduction

Microplastic particles were first found on the sea surface as early as the 1970s (Carpenter and Smith 1972) and the accumulation of plastic waste in both aquatic and terrestrial ecosystems has become nowadays one of the main environmental emergencies, considering that every year around 8 million tons of plastic end up in the ocean (Jambeck et al. 2015).

To face this problem, it is first necessary to identify and quantify abundances, sources and pathways of microplastics (Werner et al. 2017). For this reason, in recent years, microplastic waste was collected and analyzed in different environments: aquatic (Erni-Cassola et al. 2019; Schwarz et al. 2019), marine water column and sediments (Naji et al. 2017; Dai et al. 2018), marine surface waters (Cózar et al. 2014; Serranti et al. 2018), sandy beaches (Bosker et al. 2018; Tiwari et al. 2019), salt marsh habitat (Weinstein et al. 2016), lakes (Jian et al. 2020; Xu et al. 2021; Yang et al. 2022) and rivers (Ding et al. 2019; Pan et al. 2020, Zhou et al. 2021).

In particular, rivers are a major pathway of microplastics to the marine environment (Faris and Hart 1994; Allsopp et al. 2006; Guerranti et al. 2017; Lebreton et al. 2017; Schmidt et al. 2017; Li et al. 2020) due to human activities, such as industry (Karlsson et al. 2015), agriculture (Corradini et al. 2019; GESAMP 2013; Huang et al. 2021) or wastewater treatment plants (Kay et al. 2018; Conley et al. 2019; Dalu et al. 2021). It has been estimated that between 1.15 and 2.41 million tons of plastic waste enter the ocean each year from rivers (Lebreton et al. 2017), with 20 rivers (15 from Asia, 3 from Africa and 2 from South America) responsible of about 67% of the total world pollution. Despite the central role of rivers in marine microplastic pollution, less studies are dedicated to freshwater microplastics than to marine microplastics, even if they are growing in recent years (Lambert and Wagner 2018; Campanale et al. 2020).

Microplastics are defined as particles having a diameter < 5 mm (Barnes et al. 2009; Thompson et al. 2009) and they can be of primary or secondary origin. Primary microplastics are specifically produced to be of microscopic size (Cole et al. 2011), such as personal care and cosmetic products (Fendall and Sewell 2009; Praveena et al. 2018). Secondary microplastics are produced by degradation of large objects through atmospheric agents (i.e. UV solar radiation, wave action and temperature change) and other physical, chemical or biological effects (Ivar do Sul and Costa 2014). Generally, microplastics can be classified into different categories according to their shape and surface texture, correlated to their potential origin (Wu et al. 2018). Even if there is not a standard classification, the most common categories are (Löder and Gerdts 2015): pellets, hard, rounded particles of primary origin; fragments, hard, jagged-edged particles deriving from larger objects; filaments or fibers, fibrous or thin uniform plastic strands, usually coming from fishing lines or textiles; films, thin, soft 2-dimensional plastics, coming from bags or wrapping materials; foam, i.e., soft styrofoam-type material of secondary origin; granules, regular rounded particles usually smaller in size than pellets (about 1 mm in diameter), considered of secondary origin. The relative abundance of microplastic categories in global freshwaters cannot be easily defined due to the lack of a standardized protocol for their classification: different studies mention different size ranges, types and numbers of categories making difficult a quantitative comparison. According to Koelmans et al. (2019) the most frequent categories of microplastics detected in freshwaters from many sites around the world are, in descending order, fragment, fiber, film, foam and pellet.

Once microplastics are sampled and categorized based on their shape, efficient methods are needed for polymer identification. Fourier transform infrared spectroscopy (FT-IR) and Raman spectroscopy are the most common methods used to identify and characterize microplastics (Klein et al. 2018; Tirkey and Upadhyay 2021). Both techniques are non-destructive and are based on the detection of different energy absorption of the polymer functional groups. Furthermore, they can perform both single-point measurement and image mapping, applied to individual particles and, thus, require a long analytical time (Käppler et al. 2016).

Another technique applied to microplastic identification is pyrolysis–gas chromatography coupled with mass spectrometry (Py-GC–MS), a destructive method that allows the identification of polymer type by combustion and analysis of the products of thermal degradation (Klein et al. 2018); the great advantage is the sensitivity that allows to detect nanoplastics but one limitation is the thermal degradation of samples (Faltynkova et al. 2021). Finally, a recent study proposes for the first time the use of Laser Induced Breakdown Spectroscopy (LIBS) to classify microplastic particles even if the surface is contaminated or oxidated (Sommer et al. 2021). This method, based on laser ablation of sample surface, allows to extract information about the sample chemical composition and the molecular structure. This is a promising technique, nearly non-destructive due to ablation but having the problem of the matrix effect which can reduce the accuracy of quantitative measurements and in which spectra are acquired with the single shot (Junjuri and Gundawar 2020).

In recent years, hyperspectral imaging (HSI) has begun to be applied in the field of automated recognition of microplastics from marine environment (Serranti et al. 2018, 2019; Shan et al. 2018; Zhu et al. 2020). HSI is an accurate and non-destructive analytical technique that combines the advantages of spectroscopy with those of digital imaging, allowing the fast acquisition of spectra of all pixels of the image with fine wavelength resolution in different spectral ranges, depending on the application. The result of a HSI acquisition is a three-dimensional dataset, the so-called hypercube, characterized by two spatial dimensions (the investigated image area) and one spectral dimension (the spectral signature of each pixel of the image). Identification of polymers is particularly effective in the near infrared and short-wave infrared (NIR-SWIR) wavelength ranges, based on their different characteristic absorption features (combination and overtone bands).

The main advantage of HSI is that, thanks to the use of dispersive elements and sensor arrays, it is possible to investigate larger areas in less time in comparison with the currently most adopted technologies for microplastic classification, FT-IR and Raman spectroscopy. HSI is generally coupled with chemometric and multivariate statistical analysis for polymer identification through the construction and application of classification models. Model construction is the most time-consuming phase, however, the further application to the acquired images, containing many microplastic particles, is very fast, taking just a few seconds, even with a standard PC. Furthermore, image acquisitions by HSI does not require any special sample preparation. The cost of the device is lower (up to 70 k USD for a HSI benchtop system) compared to that of FT-IR and Raman imaging systems (over 200 k and 100 k USD, respectively) (Faltynkova et al. 2021). One limitation of HSI is the minimum size of detectable microplastic particles, that is usually about 60 μm, whereas FT-IR and Raman spectroscopy can detect even smaller microplastics, starting from 10–20 μm and 1–2 μm, respectively (Vidal and Pasquini 2021).

In this work, HSI was applied to the characterization of microplastics collected along the Po river (northern Italy), the main Italian river both for length (652 km) and discharge (1490 m^3^ average outflow per year 1917–2020) (Regione Emilia-Romagna 2000–2018), whose basin is an international watershed: its surface extends for 74,000 km^2^ of which about 71,000 km^2^ across Italy, which means a quarter of the entire national territory. Po river basin is home to roughly 15 million inhabitants and includes many large cities and areas of intensive industrial and agricultural activities.

To the best of our knowledge, HSI in the SWIR range (1000–2500 nm) was applied for the first time in this work to classify microplastics collected in freshwater, demonstrating its effectiveness through the implementation of a hierarchical partial least squares-discriminant analysis (PLS-DA) classification approach, allowing to simultaneously identify many different polymers.

The objectives of the study can be summarized as follows: i) to monitor microplastic pollution of the longest Italian river that crosses a relevant area, ii) to identify polymer types using HSI, a new, rapid, and non-destructive technology in this field, coupled with the implementation of a hierarchical classification approach, iii) to carry out an automatic morphological and morphometrical characterization of the identified microplastic particles (i.e. measurement of the main shape and size parameters), and iv) to compare the obtained results, in terms of abundance and composition, with those of other studies on microplastics collected along foreign and Italian rivers.

## Materials and methods

### Materials

**Fig. 1:**
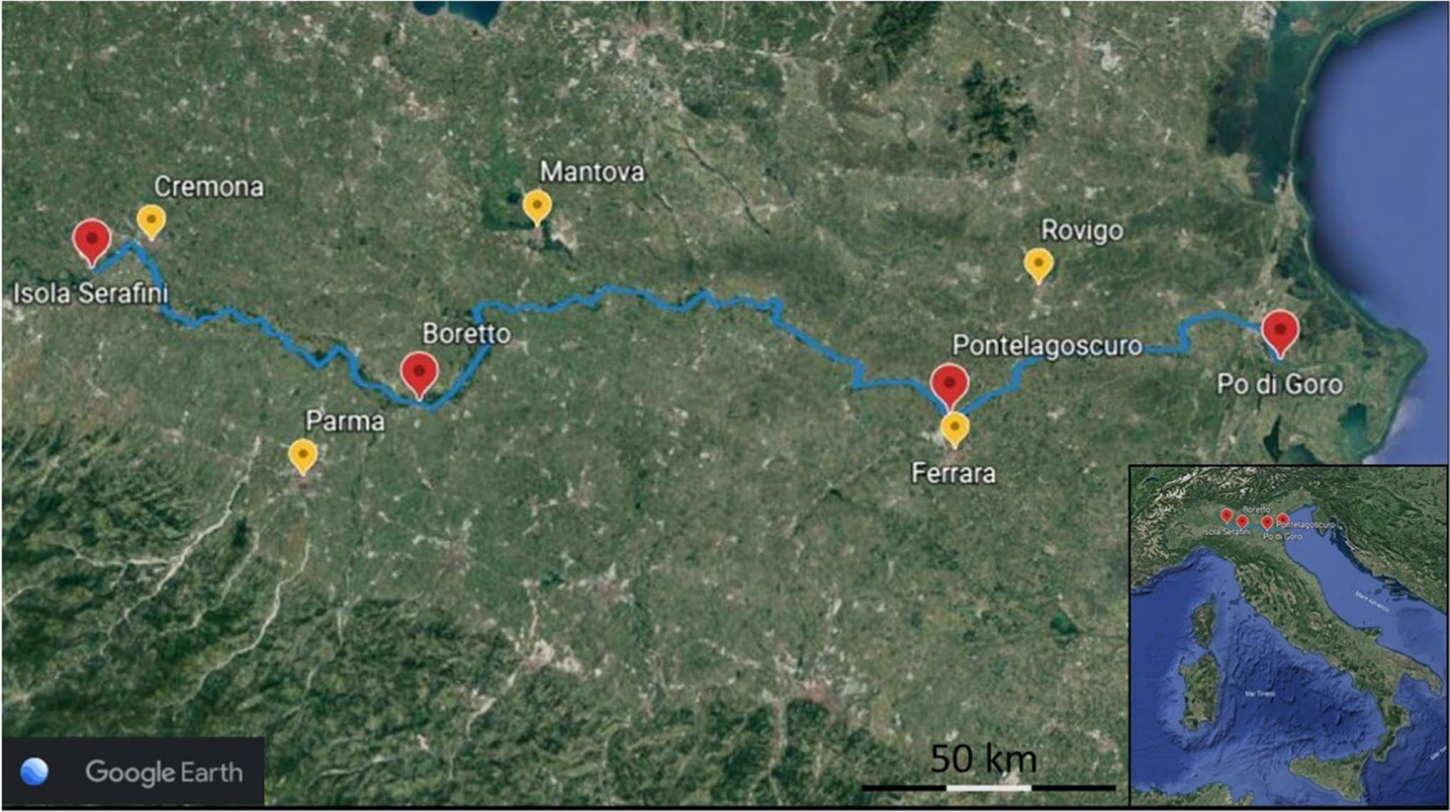
Location of the four sampling sites (Isola Serafini, Boretto, Pontelagoscuro and Po di Goro) along the Po river (Italy) indicated by red location markers. Yellow location markers indicate the Italian provinces crossed by the Po river

Microplastics were sampled by ARPAE (Regional Agency for Environmental Prevention and Energy of Emilia-Romagna) in February 2020, in the framework of the “Manta River Project” financed by “District Basin authorities of Po river,” along the Po river (northern Italy), at four different sites: Isola Serafini, Boretto, Pontelagoscuro and Po di Goro, as shown in Fig. 1.

### Methods

#### Sampling

Stated that rivers represent a source of particular interest for litter loads in marine ecosystems (Guerranti et al. 2017), the same range mesh size (> 0.3 mm < 5 mm), as the one used for the monitoring activities in the Marine Strategy Framework Directive—2008/56/EC, was adopted in this work, in order to provide comparable data among different studies related to the Adriatic Sea and the Po river.

A 333 µm manta trawl with a rectangular opening of 30 cm high by 60 cm wide was utilized. The net was towed for 20 min along the surface using small vessel keeping half of the manta net opening carefully submersed during the sampling. In this way at each sampling sites one water sample was collected.

Start–end position points were recorded from the ship’s GPS. After completion of each tow the net was washed thoroughly with freshwater from the outside of the net in order to concentrate all particles adhered to the net into the cod end. The sample collected in the cod end was transferred in glass bottle in 70% ethanol solution in order to preserve the sample which is rich in organic matter. The samples were stored into the fridge at 4 °C until subsequent analyses.

The number of microplastic particles per sample has been normalized per km^3^. The formula used for normalization is microplastic particles per sample/sampling area, where sampling area is calculated by multiplying sampling distance by the width and the height of the submerged part of the net. Sampled water volumes varied from 500 to 709.5 m^3^ (mean: 676.38 ± 119 m^3^).

The number of microplastic particles per sample has been normalized per km^3^. The formula used for normalization is microplastic particles per sample/sampling area, where sampling area is calculated by multiplying sampling distance by the width and the height of the submerged part of the net. Sampled water volumes varied from 500 to 709.5 m^3^ (mean: 676.38 ± 119 m^3^).

#### Laboratory analysis of microplastics

During the pre-treatment step, the samples were wet-sieved with distilled water using a 300 µm and 5 mm stainless steel sieve in order to obtain a water volume reduction. The microplastics particles were visually observed and separated from preserved natural material using tweezers under a stereomicroscope Nikon SMZ800 10 × 80x objective Plan Apo 1X w.d. 70 mm, circular polarizing (with cold light source Photonic PL 2000 Lumen), photographed (with attached Nikon camera DS-L2). All material longer than 5 mm was discarded. Finally, the plastic items were stored in small glass vials (Viršek et al. 2016).

Microplastics were categorized according to color (white, black, red, green, yellow, blue, other color) defining its transparency or opacity and shape. Five different shape categories were recorded: fragment, filament, pellet, foam and granule according to the Master List of Categories of Litter Items (Löder and Gerdts 2015).

In order to prevent contamination during sample processing some precautions have been respected (Hidalgo-Ruz et al. 2012; Cowger et al. 2020): during the microplastics separation, the samples when not in use was properly cover with glass in order to prevent contamination, no plastic materials or devices were used but only glass and metallic thoroughly washed three time with Milli-Qwater, 100% cotton clothes were worn and only the technician involve in the analysis was present in the laboratory.

**Fig. 2:**
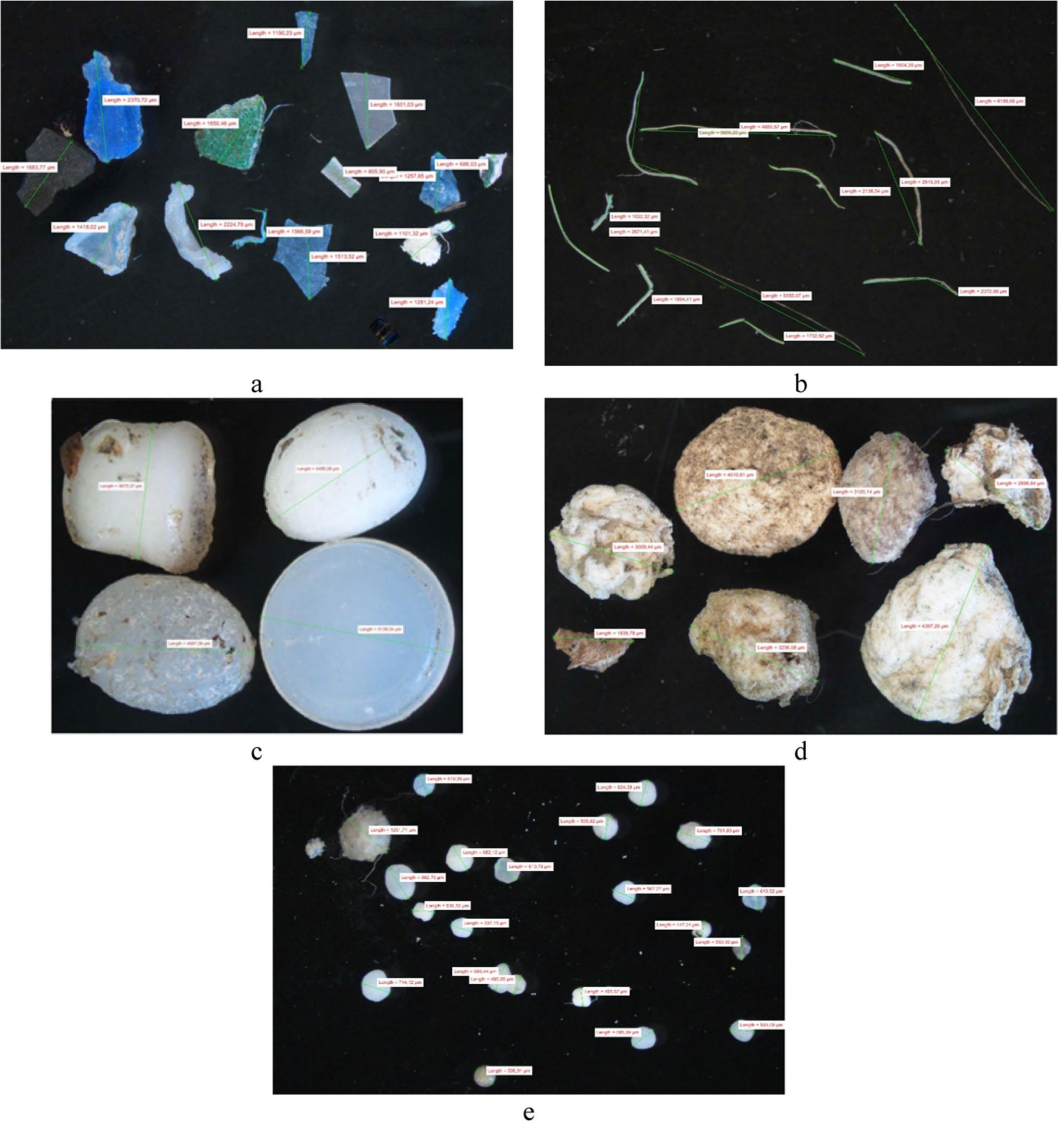
Examples of the collected microplastics: fragment (**a**), filament (**b**), pellet (**c**), foam (**d**), and granule (**e**)

In order to check for airborne fiber contamination, a control Petri dish was left open on the working table during all stages of the analyses and cross examined. On the blanks, only white fibers were found with an average of 2 per blank and the filament abundances were blank corrected. Finally, microplastics were measured and weighed. In the 4 sampling sites a total of 299 microplastic particles were collected and analyzed. Some examples of microplastics collected during the study are shown in Fig. 2.

#### Hyperspectral imaging analysis for microplastic characterization

Microplastic particles were investigated at the RawMaLab (Raw Materials Laboratory) of the Department of Chemical Engineering, Materials & Environment (DICMA), Sapienza University of Rome. Hyperspectral images of the samples were acquired by SISUChema XL™ Chemical Imaging Workstation, equipped with the spectrograph ImSpector ™ N25E (Specim, Finland) operating in the SWIR range (1000–2500 nm). The "macro" lens was used, allowing to capture a 10 mm field of view with a spatial resolution of 30 µm/pixel and an acquisition speed of 2.55 mm/s. The spectral resolution is 6.3 nm. The utilized hyperspectral system works in pushbroom mode: the HSI camera is mounted on a conveyor belt in which microplastic particles are placed and images are acquired line by line, simultaneously acquiring a full spectrum for each pixel of the line.

The corresponding digital images of the samples were acquired using a Nikon D5200 camera. In total 42 hyperspectral images and 42 corresponding digital images were acquired, divided by microplastic category for each sampling station.

For the construction of the classification model, the hyperspectral images were imported into MATLAB® environment (Version 9.3.0, The Mathworks, Inc.) and subsequently analyzed using the PLS_toolbox (version 8.6; Eigenvector Research, Inc.).

Hyperspectral images were preprocessed in order to highlight the spectral differences between different types of polymers: expanded polystyrene (EPS), polyamide (PA), polyethylene (PE), polyethylene terephthalate (PET), polypropylene (PP), polystyrene (PS), and polyvinyl chloride (PVC). Principal component analysis (PCA) was then applied for exploratory purposes, to verify the effective possibility of discriminating polymers. Finally, a hierarchical PLS-DA (HI-PLS-DA) classification model was built for the recognition of the different polymers.

##### Spectra preprocessing and principal component analysis

A fundamental step to eliminate undesirable phenomena, such as light scattering and noise, is represented by spectral preprocessing. In this study, different combinations of algorithms were used such as: Derivative, Standard Normal Variate (SNV), Normalize, Detrend and Mean Center (MC). In more detail, the Savitzky-Golay derivative (S-G) was preliminarily used to improve the signal differences. SNV algorithm was used to reduce the light scattering due to changes in environmental conditions, Normalize was used to scale and normalize the data, Detrend was applied to remove the average offset from each sample, and, finally, MC was used to center the data calculating the mean of each column and subtracting this from the column to have zero mean (Rinnan et al. 2009).

After preprocessing, PCA was applied. PCA is an unsupervised method used for reducing data size by projecting samples into a smaller subspace in which axes (principal components) are linear combinations of the original variables and are calculated to express the maximum variance. Through this method it is possible to detect spectral similarities and differences between samples observing the different grouping of pixels in the score plot based on similar spectral signatures (De Juan et al. 2009; Jolliffe 2002).

##### Hierarchical PLS-DA classification model

Microplastic classification was carried out building a HI-PLS-DA model. PLS-DA is a linear classification method for detecting sources of data variability by combining properties of partial least squares regression with classification techniques. The classification method is performed by selecting samples belonging to known classes (training sets) on which a classification rule is based. The obtained model is validated using an unknown data set (validation set) (Barker and Rayens 2003). Cross validation was performed using the Venetian Blinds method for evaluating the complexity of the predictive model and to select the number of Latent Variables (LVs) (Ballabio and Consonni 2013).

In the HI-PLS-DA hierarchical model, classification is obtained by dividing the samples into subgroups and building individual PLS-DA models for each of them. The classification of all polymers is achieved through subsequent steps, consisting of the model nodes. In each node one or more polymers are identified with respect to the others by applying a classification rule based on the choice of the most effective spectral preprocessing methods (Bonifazi et al. 2018, 2020).

**Fig. 3:**
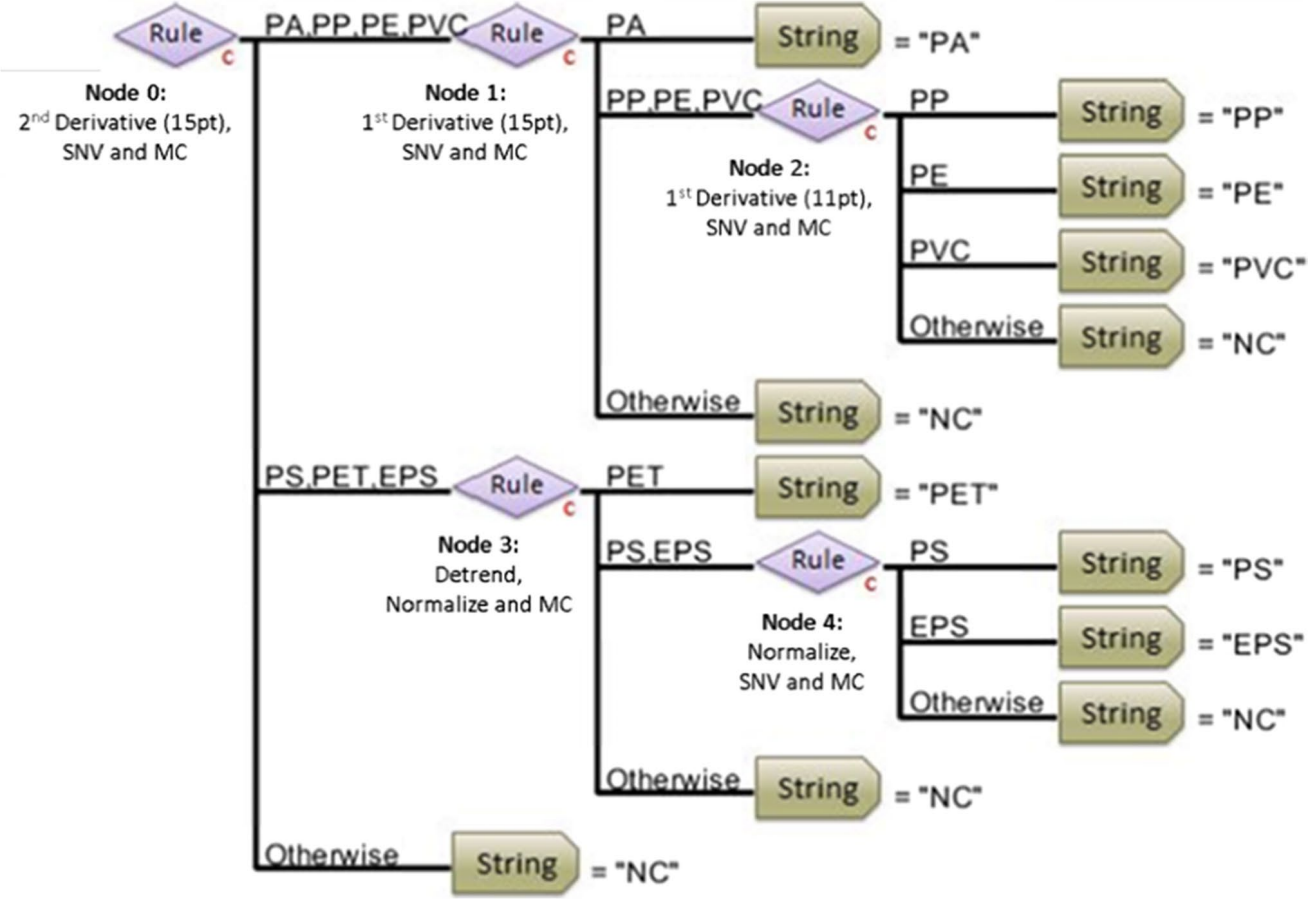
Structure of the HI-PLS-DA model built for the classification of microplastics, and spectral preprocessing algorithms applied for each node

Figure 3 shows the developed HI-PLS-DA model, which is based on five classification rules. For each rule, appropriate algorithms were chosen to highlight the different spectral signature and manage the classification problem in detail. The developed model allows to identify eight different classes, consisting of seven polymers (EPS, PA, PE, PET, PP, PS and PVC) and a “NC” class (Not Classified).

The performance of the HI-PLS-DA classification models was evaluated using two statistical parameters for both calibration (Cal) and cross-validation (CV). *Sensitivity* evaluates the model ability to avoid false negatives whereas *Specificity* to avoid false positives (Amigo et al. 2015). The values of such parameters range between 0 and 1, where 1 is the value of a perfect prediction model. More in details, *Sensitivity* and *Specificity* are expressed as (Eqs. 1 and 2):

1 $$Sensitivity=\frac{TP}{TP+FN}$$

2 $$Specificity=\frac{TP}{TP+FN}$$

where the true positive (TP) and the true negative (TN) indicate the pixels that were appropriately assigned as belonging (TP), or not belonging (TN), to a precise class. FP and FN represent false positives and false negatives, respectively, indicating the pixels that were erroneously assigned as belonging (FP), or not belonging (FN), to a precise class.

#### Morphological and morphometrical characterization

Digital images of microplastic samples were acquired using a Leica M205 C stereomicroscope with Leica 5000 series LED lighting system and equipped with an Optikam B2 camera. The magnification selected for image acquisition was 10x.

The morphological and morphometrical characterization was carried out using the Image Pro Plus software (Version 6.0.0.260, Media Cybernetics ®). For each microplastic particle, the following parameters were measured:

- *Area* (mm^2^): area of the object;
- *Aspect*: ratio between major axis and minor axis of ellipse equivalent to object. This parameter provides an indication of how much the particles are elongated;
- *Perimeter*: length of the boundary of the object;
- *Roundness*: provides an indication of the circularity of the object (for a circle the parameter takes the value 1) and is calculated with the following equation (Eq. 3):
  3 $$\frac{{\left(Perimeter\right)}^{2}}{4\pi Area}$$
- *Minimum Feret Diameter* (mm): smallest caliper (Feret) length;
- *Maximum Feret Diameter* (mm): longest caliper (Feret) length;
- *Average Feret Diameter* (mm): average caliper (Feret) length;

For each parameter, the following statistical measurements were calculated: average, standard variation, and minimum and maximum values. Furthermore, the frequency distribution plots of *Maximum Feret diameter* for microplastic categories and polymer type were built and evaluated.

The procedure carried out for the morphological and morphometrical characterization is composed by four main phases: spatial calibration, image binarization, particle counting&labeling, and morphological and morphometrical measurements. The first phase consists in the spatial calibration of the image by converting pixels into millimeters. The second phase is the binarization of the image to obtain black microplastic particles on a white background; this is necessary to carry out, in the third phase, particle counting and labeling. Finally, in the fourth phase, selected morphological and morphometrical parameters are measured. Data obtained in terms of sizes, shapes and categories were analyzed and correlated to polymer type.

## Experimental results

### Microplastic concentrations

The average abundance was 5.87 particles/m^3^ equivalent to 0.88 particles/m^2^, with minimum value of 1.89 particles/m^3^ (Isola Serafini) and maximum value of 8.22 particles/m^3^ (Boretto). Similar abundances were found in the other two sites, a concentration of 6.52 particles/m^3^ was detected at Pontelagoscuro and 6.85 particles/m^3^ at Po di Goro. The daily outflow was 653 m^3^/s at Isola Serafini, 882 m^3^/s at Boretto and 1056 m^3^/s at Pontelagoscuro (Arpae Idro-Meteo-Clima, Annali Idrological 2019). Besides the flow rate, considering that sampling was carried out in different stations, there are many other factors and variables affecting microplastics abundance and movement in the riverine system: weather conditions, hydrology (flow conditions and daily discharge), morphology (vegetation pattern), watercourse obstructions such as groynes and barrages (Lambert and Wagner 2018).

The division by category of collected microplastics showed that the more represented categories were fragment (44%) and foam (29%), followed by granule (16%), pellet (8%) and filament (3%). In particular, the fragments were predominant at Isola Serafini (63%), Boretto (45%) and Po di Goro (54%), whereas at Pontelagoscuro fragment (30%) was the second category after foam (36%). The filaments were always the category less represented in all the stations: Isola Serafini (21%), Boretto (1.4%), Po di Goro (1.8%), Pontelagoscuro (2.7%). At Isola Serafini the pellet and foam categories were not detected. Concerning the possible sources of microplastics, fragment, foam and granule microplastic categories, being of secondary origin, indicate their fragmentation from larger items probably started in land environment and their transportation by surface runoff. On the contrary, the scarce presence of pellets, being of primary origin, may indicate the adoption of correct policies by the local industries to avoid their spillage in the environment, in agreement with what observed by Munari et al. (2021). The lower presence of filaments, usually come from fishing nets, clothing or other textiles, in comparison to the other microplastic categories, could be justified considering that major losses can happen when using surface tow nets with coarse meshes. In fact, their fibrous shape, characterized by a minimum diameter smaller than the size of the adopted sampling manta net, makes difficult their collection (Ryan et al. 2020).

Overall, regarding the color, white is the dominant color (88%), more precisely opaque white (51%) and transparent white (37%). Among the sites, opaque white was the dominant microplastics color, with the exception of Pontelagoscuro site in which transparent white was prevailing. In all dataset the other colors found in descending order were blue, black, green, red.

### Hyperspectral imaging analysis

#### Spectra characterization and PCA

**Fig. 4:**
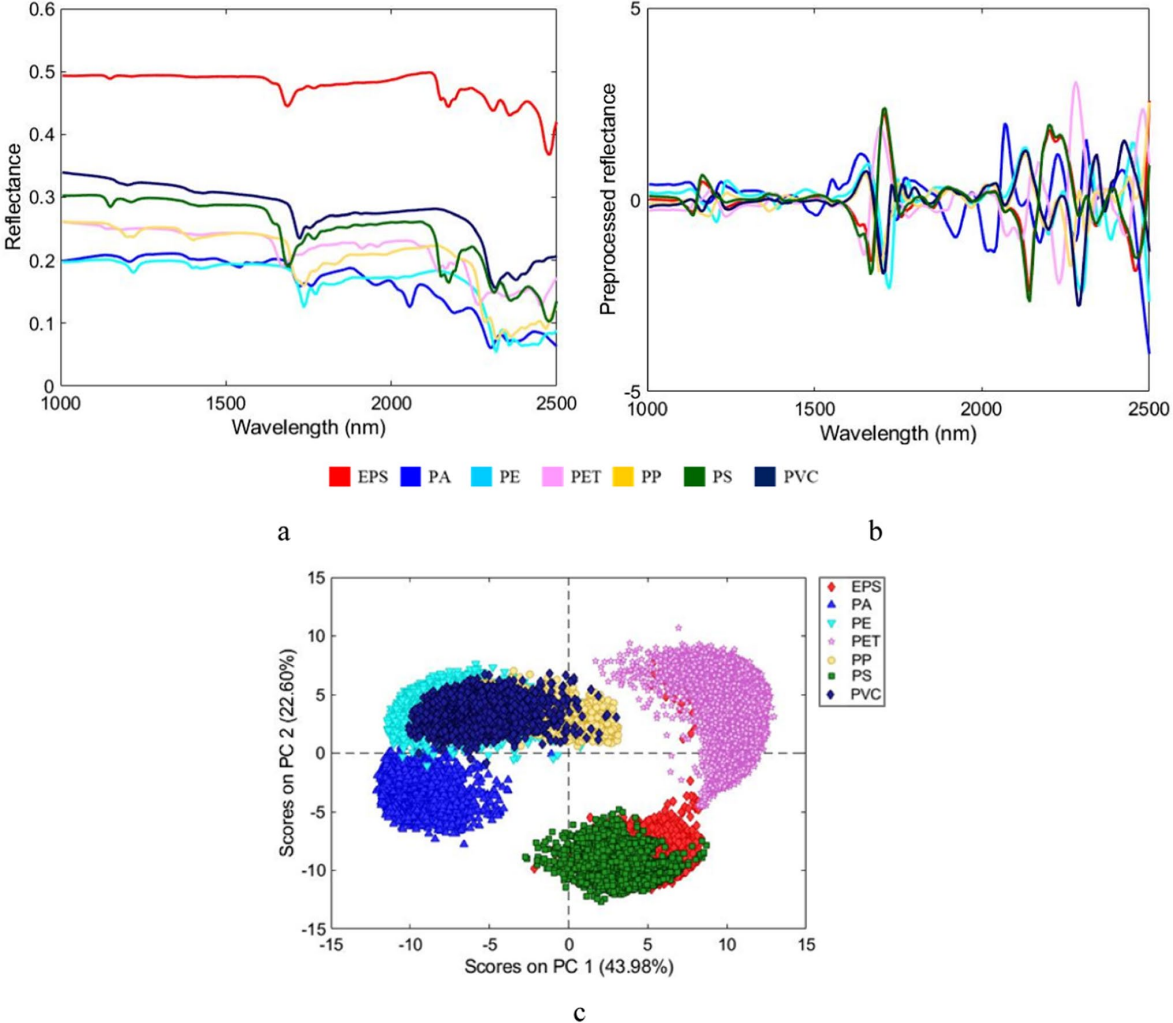
Raw average reflectance spectra acquired by HSI in SWIR range (1000–2500 nm) (**a**), corresponding preprocessed average spectra (**b**) and PCA score plot (**c**) of reference polymers (EPS, PA, PE, PET, PP, PS and PVC) used for the construction of the HI-PLS-DA model

The raw average reflectance spectra of reference polymers (EPS, PA, PE, PET, PP, PS, and PVC), the corresponding preprocessed spectra and the PCA score-plot of the training dataset are reported in Fig. 4. As shown in Fig. 4a, polymers show different spectral signatures in the SWIR range depending on variation of the overtone bands of the fundamental groups containing O–H, C–H, N–H and C–O bonds. The identification of polymers in 1000–2500 nm range is mainly based on the stretching vibration modes of CH, CH_2_ and CH_3_ groups between second and first overtone region and the first combination bands (Workman and Weyer 2012; Stuart 2004). The application of selected pre-processing algorithms (2^nd^ Derivative, SNV and MC) allowed to better highlight spectral differences between polymers (Fig. 4b). PCA score plot (Fig. 4c) indicates that most of the variance was captured by the first two principal components, which explain 44% and 23% of the variance, respectively. The spectral data of polymers are clustered in two different regions of the score plot based on the similarities/differences of the spectral signatures. More in details, PCA shows that PC1 negative values identify PA, PP, PE, and PVC, whereas PC1 positive values identify PS, EPS, and PET samples. Due to the complexity of data, clearly shown by the score plot, in which there is overlapping of pixels belonging to different polymers, a hierarchical model to simplify the classification problem was developed. In this perspective PCA score plot of Fig. 4c is representative of Node 0 of the HI-PLS-DA model.

The average preprocessed spectra of polymers and the corresponding PCA score plot for the other nodes of the HI-PLS-DA model are reported in Figure S1 of the Supplementary Material. For each rule, most of variance was captured by the first two principal components (PC1-PC2). As shown in the four PCA score plots, the spectral data of polymer samples are clustered into distinct clusters according to their spectral signatures. In more detail, in Node 1 the positive values of PC1 for PA allows its separation from the other plastics, in Node 2 it is possible to identify PP, PE and PVC samples, being clustered in different regions of the score plot, in Node 3 PET can be identified from EPS and PS based on PC1 values and, finally, in Node 4-pixel clusters of EPS and PS are clearly separated in the score plot.

#### Microplastic classification by HI-PLS-DA

**Fig. 5:**
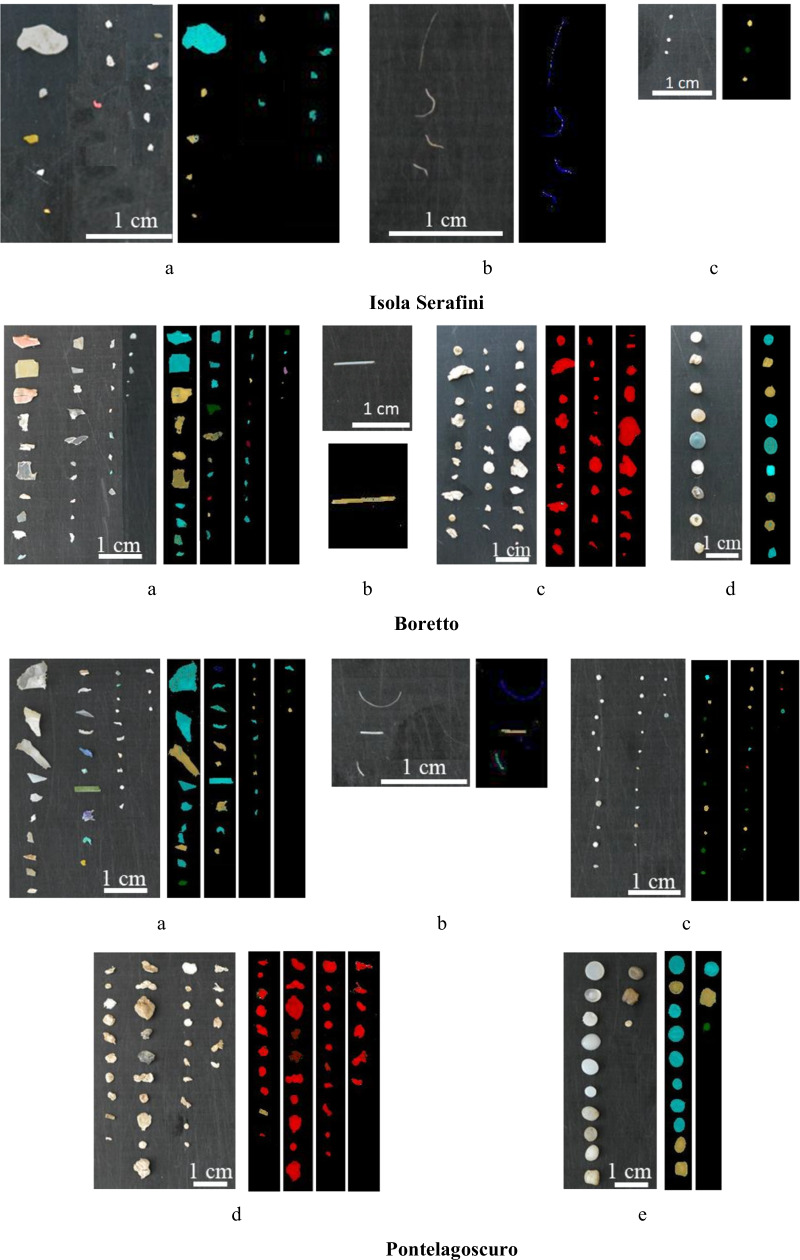

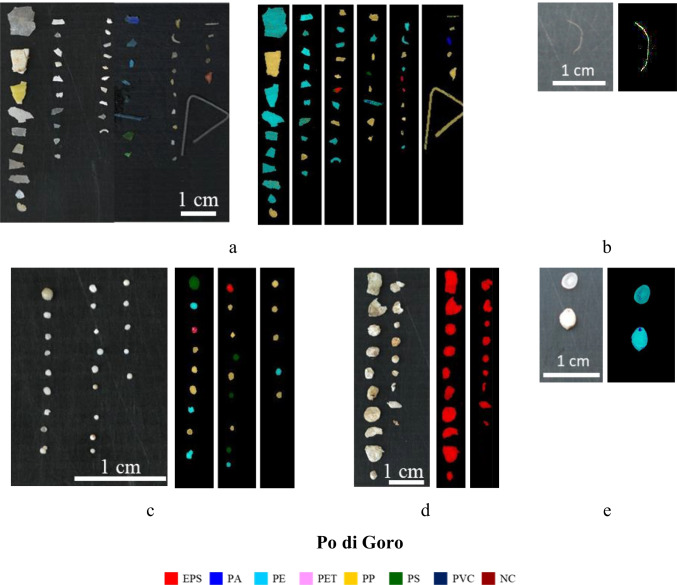
Digital images and corresponding predicted images obtained by the application of the HI-PLS-DA classification model on microplastic particles collected at Isola Serafini, Boretto, Pontelagoscuro and Po di Goro stations, belonging to fragment (**a**), filament (**b**), granule (**c**), foam (**d**) and pellet (**e**) category, respectively

The classification results of microplastic particles obtained by the application of the HI-PLS-DA model based on seven different polymer classes are reported in Fig. 5 in terms of predicted images for each microplastic category collected in each sampling station.

*Sensitivity* and *Specificity* values in calibration and cross-validation for the different rules of the hierarchical model range from 0.996 to 1.000 for both parameters confirming the very good performance of the classification model. All the values are reported in Supplementary Material.

**Fig. 6:**
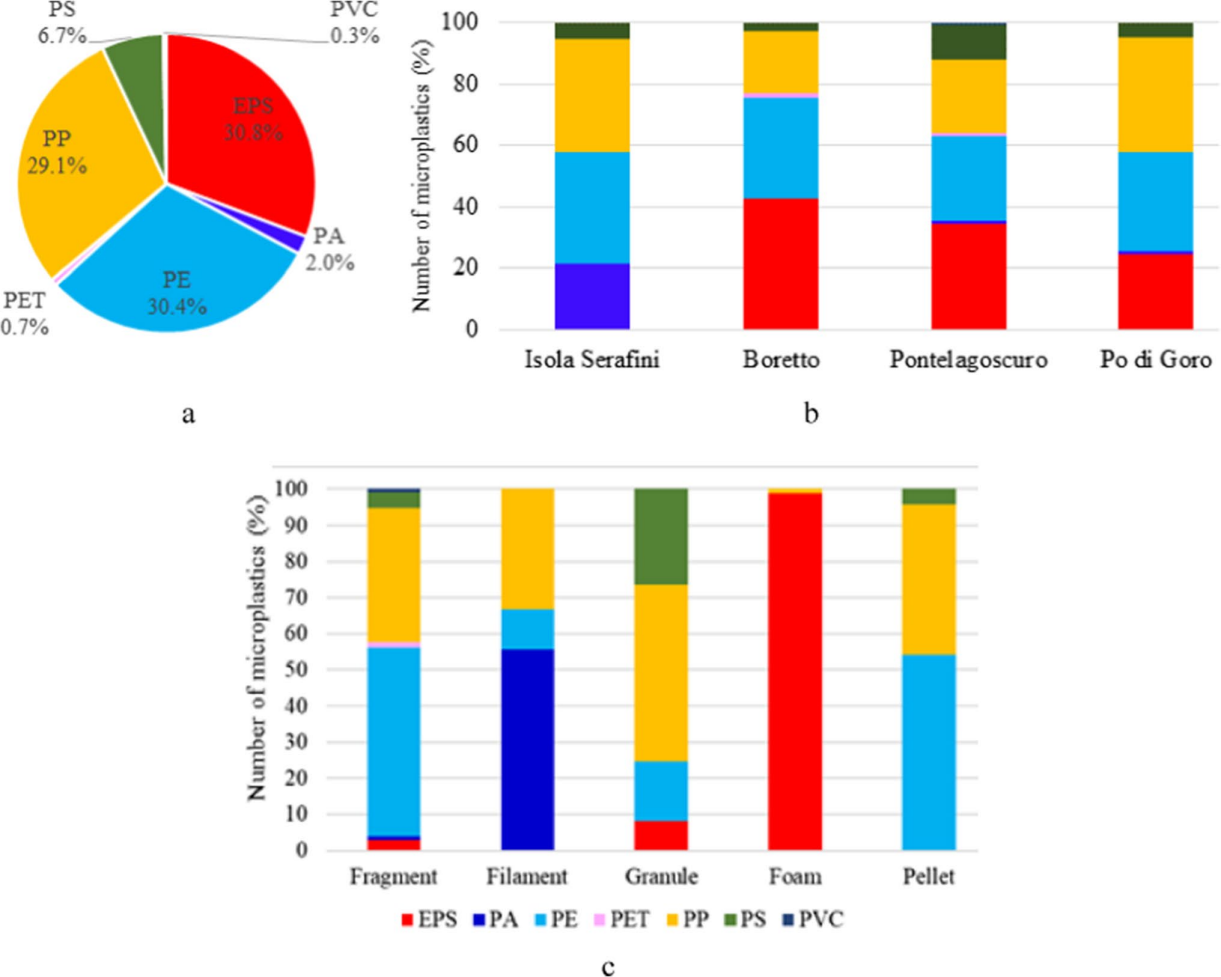
Polymer type overall abundance (**a**), polymer type distribution in each sampling station (**b**) and polymer type distribution for the different microplastic categories (**c**). All data are reported in number of microplastics (%)

The characterization of microplastics in terms of overall polymer type abundance, amount of polymer types in each sampling station and correlation between polymer type and corresponding microplastic category are shown in Fig. 6.

As shown in Fig. 6a, microplastic particles are mainly composed of EPS (30.8%), PE (30.4%) and PP (29.1%), followed by PS (6.7%) and PA (2.0%). The presence of these polymers is in agreement with their density values, being lower than that of water. The other identified polymers represent a total fraction equal to 1%: PET (0.7%) and PVC (0.3%).

From Fig. 6b, it can be noticed as in Boretto, Pontelagoscuro and Po di Goro stations the most abundant polymers are: EPS (Boretto: 42.5%; Pontelagoscuro: 34.3%; Po di Goro: 24.2%), PE (Boretto: 30.1%; Pontelagoscuro: 27.8%; Po di Goro: 32.3%) and PP (Boretto: 23.3%; Pontelagoscuro: 24.1%; Po di Goro: 37.4%), followed by PS (Boretto: 2.7%; Pontelagoscuro: 11.1%; Po di Goro: 5.1%). In Boretto and Pontelagoscuro stations the order of abundance of the polymers is the same (EPS, PE, PP and PS) although with some variations in percentages, especially of EPS and PS. In microplastic samples from Po di Goro station, however, the order of abundance is reversed compared to that observed in the two previous stations (PP, PE and EPS always followed by PS). On the contrary, in microplastic samples collected at Isola Serafini station, a different composition is observed: the most abundant polymers are PE and PP (both with 36.8%), followed by PA (21.1%) and PS (5.3%), whereas EPS was not detected. The reason can be explained by the greatest abundance of PA filaments collected at Isola Serafini station (see Paragraph 3.1), probably indicating a secondary origin from textile. PET and PVC constitute a negligible fraction: the first was detected only at Boretto and Pontelagoscuro stations (1.4% and 0.9%, respectively). As regards PVC, only one particle was identified at Pontelagoscuro station, corresponding to 0.9%.

Concerning the distribution of polymers among the microplastic categories (Fig. 6c), it is possible to note that fragments, a microplastic category of secondary origin, are mainly constituted by PE and PP (55.3% and 34.8%, respectively), followed by PS (4.5%), EPS (3.0%) and PA, PET and PVC (0.8% each). This result can be explained considering that PE and PP are the most demanded polymers in the market, especially for packaging products, that are easily dispersed in the environment, representing about 50% of global plastics production (Plastics Europe 2020). PS and EPS are largely used in food packaging, especially for dairy and fishery products. Furthermore, these polymers are characterized by a density lower than that of water, floating on the water surface and easily carried by currents (Hidalgo-Ruz et al. 2012). Filaments are constituted mainly by PA (55.6%), followed by PP (33.3%) and PE (11.1%). This category is usually of secondary origin, deriving from the degradation of nylon ropes, fabrics and fishing lines. Granules have a heterogeneous composition, being mainly composed by PP (49.0%) followed by PS (26.5%), PE (16.3%) and EPS (8.2%). They can also be considered secondary microplastics. Microplastics belonging to the foam category are composed almost only by EPS (98.8%) and are probably due to the degradation of domestic packaging and/or box used in fishing activities. The polymers constituting pellets, which are considered primary microplastics, are mainly PE (54.2%) and PP (41.7%), followed by PS (4.2%). This result, as already observed for fragments, agrees with the fact that PE and PP are the two polymers most used by industries.

### Morphological and morphometrical characterization

In the following, the most relevant results related to the morphological and morphometrical analysis are highlighted. Full data are reported in Table S2 of Supplementary Material for each detected microplastic category, in terms of minimum, maximum, mean and standard deviation of the investigated parameter.

The results obtained for the fragment category highlight a great variability for most size and shape parameters (*Area* ranged from 0.16 to 24.58 mm^2^ and *Perimeter* from 1.30 to 21.02 mm). Furthermore, they are, as expected, less rounded than pellets and granules (*Aspect* from 1.06 to 7.89 and *Roundness from* 1.00 to 3.46). The results for filaments are in agreement with the elongated shape of this category of microplastics, being characterized by small *Areas* (from 0.19 to 3.58 mm^2^) and large *Perimeters* (from 4.43 to 20.50 mm). Granule category shows a reduced variability of measured parameters, in agreement with its small size and round shape. Granules are on average the smallest (*Area*: 0.67 ± 0.47 mm^2^ and *Maximum Feret Diameter*: 0.94 ± 0.29 mm) and have a “rounded” shape, as shown by the *Roundness* and *Aspect* values (1.00–1.14 and 1.01–1.93, respectively). Some variability in shape and size parameters is evident for foam microplastics (*Aspect* ranged from 1.04 to 2.75 and *Area* ranged from 0.26 to 14.39 mm^2^). Finally, pellets show, as expected, homogeneous size and shape, being well rounded (*Roundness* values ranging from 1.00 to 1.10), according to their primary origin, but greater in size than granules (*Area*: 2.01 ± 1.31 mm^2^ and *Maximum Feret Diameter*: 1.67 ± 0.53 mm).

**Fig. 7:**
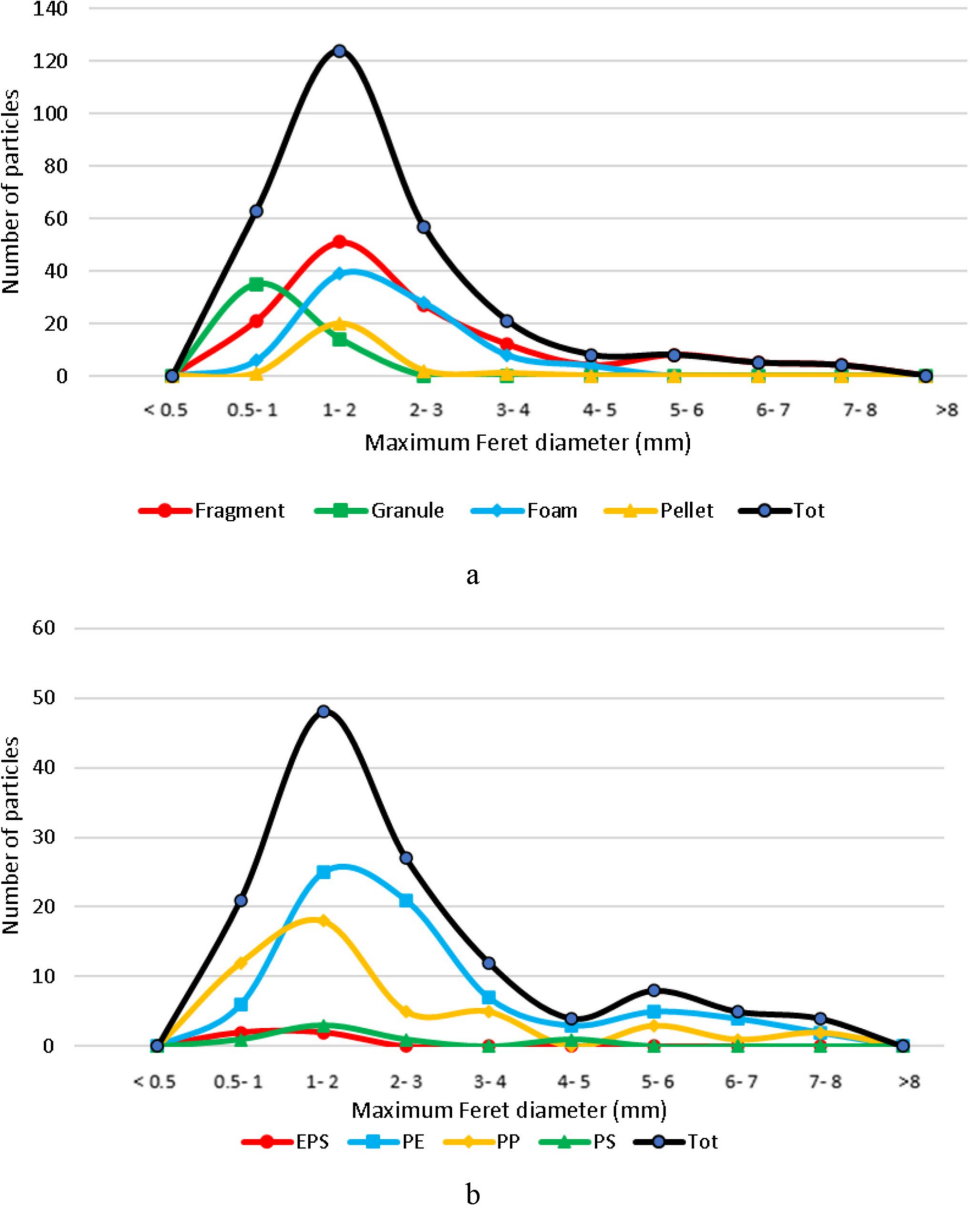
Frequency distribution (in number of particles) of *Maximum Feret diameter* for microplastics belonging to the fragment, granule, foam and pellet category (**a**) and frequency distribution (in number of particles) of *Maximum Feret diameter* for fragment category divided by polymer type (**b**). The curve in black indicates the size distribution of all particles

The size class distribution of microplastic particles divided by category, in terms of *Maximum Feret Diameter*, is reported in Fig. 7a showing that most of them are characterized by diameter values less than 5 mm, in agreement with the standard size defined for microplastics. In more detail, 87% of fragment particles have a diameter less than 5 mm whereas all granule, foam and pellet particles are smaller than 5 mm. Fragment category shows the widest size distribution, with particles in all the size classes from < 0.5 to > 8.0 mm, followed by foam category, with particles from < 0.5 to 5 mm. Pellet and foam categories show a more uniform size class distribution, ranging from 0.5 to 4 mm and 0.5 and 3.0 mm, respectively. The mode of the frequency distribution is 1–2 mm for fragment, foam and pellet particles, whereas for granules is smaller, as expected, being 0.5–1.0 mm. Concerning the mean of particle size distribution, fragments (132 particles) show the greatest value (2.42 mm), followed by foam (85 particles) with an average value of 2.07 mm, pellet (24 particles) with an average value of 1.67 mm and granule (49 particles) with an average value of 0.94 mm.

Concerning fragments, the most abundant microplastic category, further considerations were made in terms of frequency distribution of *Maximum Feret Diameter* divided per polymer type considering only the most abundant ones (i.e. EPS, PE, PP and PS) (Fig. 7b). From the graph it can be noticed as the mode of the size class distribution is 1–2 mm for fragment particles of all polymer types. PE and PP particles are characterized by the greatest size variability, ranging from 0.5 to 8 mm, PS particles range from 0.5 to 5 mm, whereas EPS fragments show the smallest and most homogeneous particle size, being distributed equally among 0.5–1 mm and 1–2 mm classes. Concerning the mean of particle size distribution, PE (69 particles) shows the greatest value (2.70 mm), followed by PP (49 particles) with an average value of 2.23 mm, PS (6 particles) with an average value of 1.95 mm and EPS (4 particles) with an average value of 1.19 mm. The size distribution of fragments belonging to different polymers, that are microplastics of secondary origin, could be correlated to their different degradation behavior. Taking into account only PP and PE, being the most abundant polymers in fragment category, the results suggest a fragmentation in smaller particles for PP with respect to PE. This result is in agreement with what observed in a previous study on microplastics sampled in marine waters (Serranti et al. 2018) and in several studies related to artificial degradation of polymers, showing that the fragmentation rate is higher for PP than for PE when exposed to UV rays (Cai et al. 2018; Song et al. 2017). In fact, PE is characterized by higher crystallinity values than those of PP (Lambert and Wagner 2018) suggesting that its more complex and ordered structure counteracts degradation.

## Comparison with other studies on microplastics collected along foreign and Italian rivers

Microplastic concentrations measured along Po river in this work were first compared with those found in rivers from different areas of the world. In most cases, concentrations were higher than those detected in Po river. However, it must be taken into account that the comparison of measured concentrations can be affected by different sampling and analytical strategies used in different studies. River contamination varies between sampling sites, ranging from values close to a few tens to a few thousand of fragments/m^3^.

More in details, in China, values equal to 10,200, 4100, and 0.7 fragments/m^3^ were detected at Yangtze River Estuary, Minjiang Estuary and Pearl River (Zhao et al. 2014; Zhao et al. 2015; Mai et al. 2019), respectively. In Africa, the highest abundance of microplastics were from South-eastern bays of South Africa having 1215 particles/m^3^, whereas in the Australian estuarine areas microplastics achieved average values exceeding 1000 fragments/m^3^ (Hitchcock and Mitrovic 2019). In Brazil (Guanabra) and in North America (Chicago Metropolitan Area) microplastic concentrations of 21.3 fragments/m^3^ (Olivatto et al. 2019) and 5.7 fragments/m^3^ (McCormick et al. 2016) were detected, respectively.

Microplastic concentrations measured in the main European rivers are comparable, with some variations, to those measured in the present study: Seine (1.7–37.7 particles/m^3^, Alligant et al. 2019; 3–108 particles/m^3^, Dris et al. 2015), Thames (14.2–24.8 particles/m^3^, Rowley et al. 2020), Rhone (0.3–59 particles/m^3^, Constant et al. 2020), Ebro (1.95–4.3 particles/m^3^, Simon-Sánchez et al. 2019), Rhine (1.85–4.92 particles/m^3^, Van der Wal et al. 2015), Danube (10.6 particles/m^3^, Van der Wal et al. 2015) and Meuse – Dommel (67–11.532 particles/m^3^, Mintening et al. 2020). The French coastal river Têt (located in the Eastern Pyrenees) showed higher concentration values, equal to 618 fragments/m^3^ (Constant et al. 2020).

Few studies have been carried out on microplastic occurrence in Italian rivers, collected from both freshwaters and sediments. The results of these studies are summarized and compared with those of this work in Table 1, in terms of sampling origin, concentration, mesh size and/or sampling volume and depth, particle size ranges, identified categories, and polymer types. The comparison of data among different studies is currently hindered by the enormous variety of adopted methodologies in all the steps of microplastic analysis, such as field sampling, preparation, identification, categorization, quantification (Cowger et al. 2020).

**Table 1:** Summary of the main studies carried out on microplastic occurrence in Italian rivers in terms of sampling origin (sediment or water), investigated particle size ranges, most abundant microplastic category, microplastic concentrations, polymer types and measurement method

Location	Sampling origin	Microplastic concentrations	Mesh size/sampling volume and depth	Particle size ranges	Most abundant microplastic category	Measurement method and polymer types		Reference
Po river (northern Italy)	River surface	1.89 – 8.22 particles/m^3^	Manta trawl 333 µm	0.50–7.84 mm	Fragments (44%), foams (29%), granules (16%), pellets (8%) and filaments (3%)	Method: HSI EPS (30.8%), PE (30.4%), PP (29.1%), PS (6.7%), PA (2.0%), PET (0.7%) and PVC (0.3%)		Present study
Po river (northern Italy)	River surface	0.29 – 3.47 particles/m^3^	Hydro-Bios Manta trawl 300 µm	80.6% with dimension < 5 mm	Fragments (67%), fibers (30%) and pellets (3%)	Method: FT-IR spectroscopy PE (40.5%), PP (25.7%), PS (14.9%), PET (8.1%), PVC (5.4%), PA (4.1%) and EVA (1.3%)		Munari et al. 2021
Po river (northern Italy)	Waters and beach sediment	Waters: 1–84 particles/m^3^ Beach sediment: up to 78 particles/DW kg	Mini-manta trawl 300 µm	1–5 mm	-	Method: FT-IR spectroscopy PE, PS and PP		Atwood et al. 2019
Po River delta (northern Italy)	Beach sediments	2.92—23.30 particles/DW kg	Stainless steel frame (25 × 25 cm), upper 5 cm was extracted	1–5 mm	Fragment (95.0%)	Method: FT-IR spectroscopy PE, PS and PP	Piehl et al. 2019	
Po River (northern Italy)	River surface	14.6 particles/m^3^	Manta Net 330 µm	< 5 mm	Fragment	Method: ATR FTIR and NIR PE (75%), PP (17%), PS (4%) and others	Van der Wal et al. 2015	
Cecina river estuary (Tuscany, central Italy)	Sediments from the coastal area	72—191 particles/DW kg	5 cm of depth in wide 1 L glass jars	< 5 mm	Fragment, fiber and granule	-	Blašković et al. 2018	
Ombrone river (Tuscany, central Italy)	Sediments samples	45—1069 particles/DW kg	50 cm of depth in 2 L bucket	0.5–10 mm	Filament and fragment	-	Guerranti et al. 2017	
Ofanto river (southeast Italy)	River surface	0.9 ± 0.4 – 13 ± 5 particles/m^3^	Plankton nets 333 µm and an opening of 55 × 55 cm	300–5000 µm	Fragment (56%) and flake (26%)	Method: Py-GC–MS PE (76%), PS (12%), PP (10%), PVC (0.7%) and TDI-PUR (0.35%)	Campanale et al. 2020	

*DW* dry weight; *EPS* expanded polystyrene; *PE* polyethylene; *PP* polypropylene; *PS* polystyrene; *PA* polyamide; *PET* polyethylene terephthalate; *PVC* polyvinyl chloride; *TDI-PUR* polyurethane; *Py-GC–MS* pyrolysis gas chromatography mass spectrometry; *HSI* hyperspectral imaging; *ATR FT-IR* attenuated total reflection Fourier transform infrared spectroscopy; *NIR* near infrared spectroscopy.

Microplastic concentration ranged from 0.29 to 84 particles/m^3^ in water and from 2.92 to 1069 particles/DW kg in beach sediment. Studies related to microplastics collected in Po river water show values lower (0.29–3.47 particles/m^3^, Munari et al. 2021) and higher (14.6 particles/m^3^, Van der Wal et al. 2015; 1–84 particles/m^3^, Atwood et al. 2019) than those measured in the present study. Furthermore, Po river water microplastic concentrations are comparable to those detected in Ofanto river (from 0.9 ± 0.4 to 13 ± 5 particles/m^3^, Campanale et al. 2020). Different sampling methods were used in the studies, as can be seen from Table 1: the studies carried out on the river surface used a manta net with mesh sizes ranging between 300 µm (Munari et al. 2021; Atwood et al. 2019), 330 µm (Van der Wal et al. 2015) and 333 µm (present study, Campanale et al. 2020); the sediment samples were taken at a depth of 5 cm in the studies of Piehl et al. (2019) and Blašković et al. (2018) and up to 50 cm in the study of Guerranti et al. (2017).

In the studies on Italian rivers, microplastics have on average a size smaller than 5 mm in water and/or sediments, except in the study by Guerranti et al. (2017), in which samples reached 10 mm, in the study by Munari et al. (2021) and in the present study, in which 87.1% and 80.6% of the collected plastics has a size < 5 mm, respectively). The most abundant microplastic category is fragment in all the studies, including those related to Po surface waters, according to its secondary origin from the degradation of larger plastic waste dispersed in the environment.

Different analytical methods were used to perform polymer identification: HSI (present study), FT-IR (Munari et al. 2021; Atwood et al. 2019; Piehl et al. 2019), FT-IR and NIR spectroscopy (Van der Wal et al. 2015) and Py-GC–MS (Campanale et al. 2020). The advantages and limitations among techniques were discussed in the Introduction, in any case the great variety of techniques suggests the need to define standardized methods for microplastic investigation in order to produce more comparable data.

In all studies carried out along Po river, including this work, the most abundant polymers are always PP, PE and PS (in our case the latter is subdivided in PS and EPS), with some variation in the order of abundance, probably depending on several factors, related to both sampling location and analytical methods.

## Conclusions

In this study, freshwater microplastics collected along the Italian Po river were characterized by developing and implementing a hierarchical PLS-DA classification model applied to hyperspectral images acquired in the SWIR range. In addition to the polymer type identification, abundance, categories and morphological and morphomerical parameters of microplastic particles from the four different sampling stations were defined and compared. The data achieved in terms of concentration constitute a solid estimate of the microplastics abundance in the Po river. It is important to consider that these data can be influenced by environmental factors such as flow rate, weather conditions**,** run-off phenomena and the intrinsic diversity of the sampling locations.

Seven different types of polymers were identified by HSI: the most abundant are EPS, PE, PP, and PS, as expected by their density which is lower than that of water, followed by PA, PET and PVC. These polymers are the most diffused in the market, most of them especially as packaging materials. The results of morphological and morphometrical characterization of microplastics are consistent with the classification in categories. Furthermore, most of the collected microplastics have a maximum Feret diameter less than 5 mm and the most populated size class is between 1 and 2 mm. Finally, the comparison among the particle sizes of the two most abundant polymers in fragment category shows that on average PE microplastics are larger than PP microplastics, suggesting a different fragmentation behavior probably due to the polymer properties, such as density and crystallinity.

Overall, the results show that the application of HSI on freshwater microplastics can be considered as an emerging suitable method for the characterization and classification of samples in a rapid, reliable and non-destructive way. Its features are very promising and useful for monitoring microplastic pollution of rivers, oceans, and coasts, contributing to the definition of the best waste management strategies.

## Supplementary Information

Below is the link to the electronic supplementary material.

Supplementary file1 (PDF 294 KB)

## Data Availability

The datasets used and/or analyzed during the current study are available from the corresponding author on reasonable request.
