# Supplementary material for: Classification and distribution of freshwater microplastics along the Italian Po river by hyperspectral imaging
Source: Environ Sci Pollut Res Int. 2022 Feb 23;29(32):48588–606. doi: 10.1007/s11356-022-18501-x (PMC9252960; doi:10.1007/s11356-022-18501-x)
Supplement: Supplementary file 1 — Supplementary file1 (PDF 294 KB) [file 11356_2022_18501_MOESM1_ESM.pdf]

Classification and distribution of freshwater microplastics along the Italian Po river by hyperspectral imaging

Environmental Science and Pollution Research

Ludovica Fiore, Silvia Serranti*, Cristina Mazziotti, Elena Riccardi, Margherita Benzi, Giuseppe Bonifazi

***** Corresponding author: Silvia Serranti, Department of Chemical Engineering, Materials & Environment, Sapienza University of Rome, Via Eudossiana 18, 00184, Rome, Italy.
email address: silvia.serranti@uniroma1.it

Supplementary Material

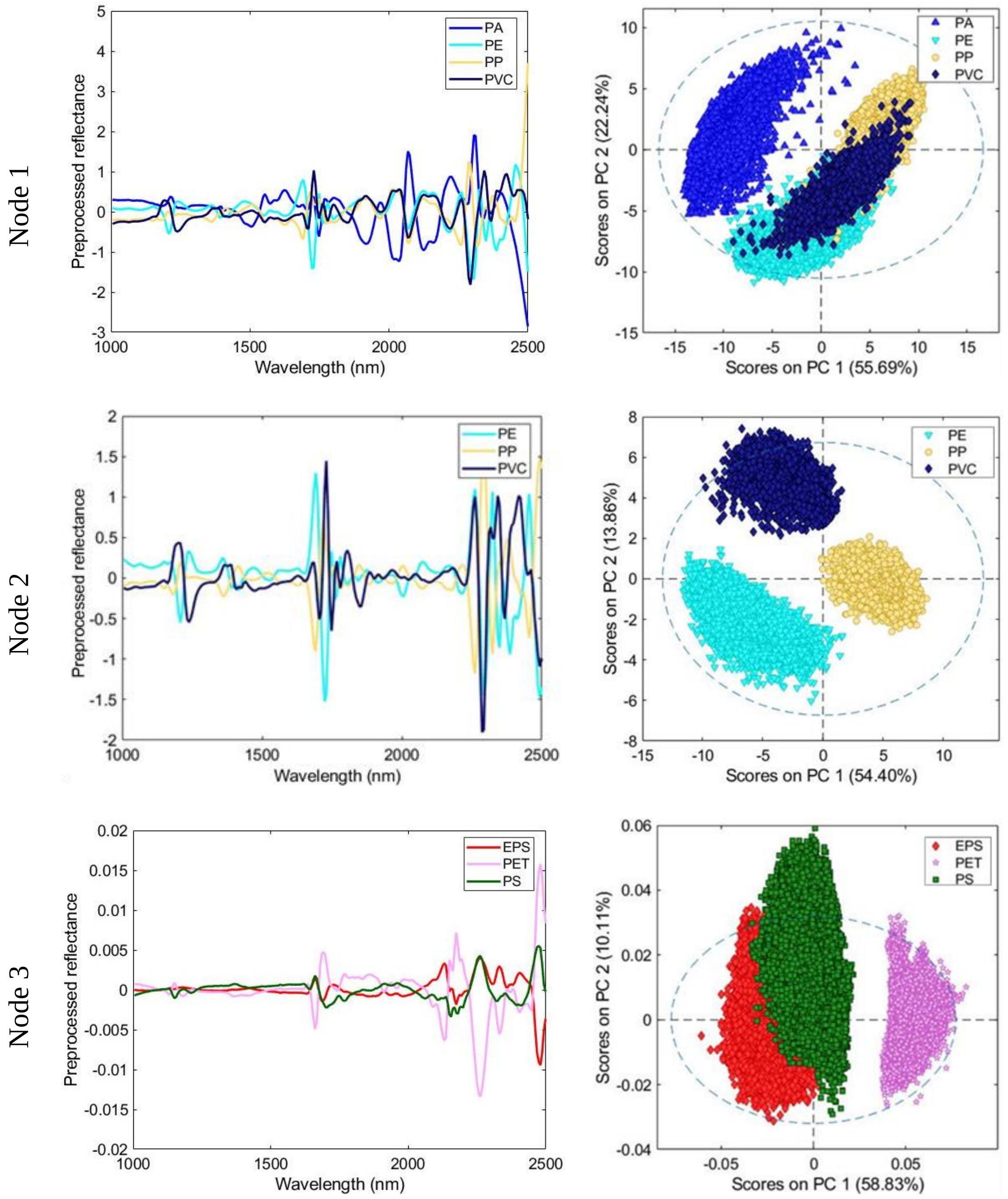

Node 4

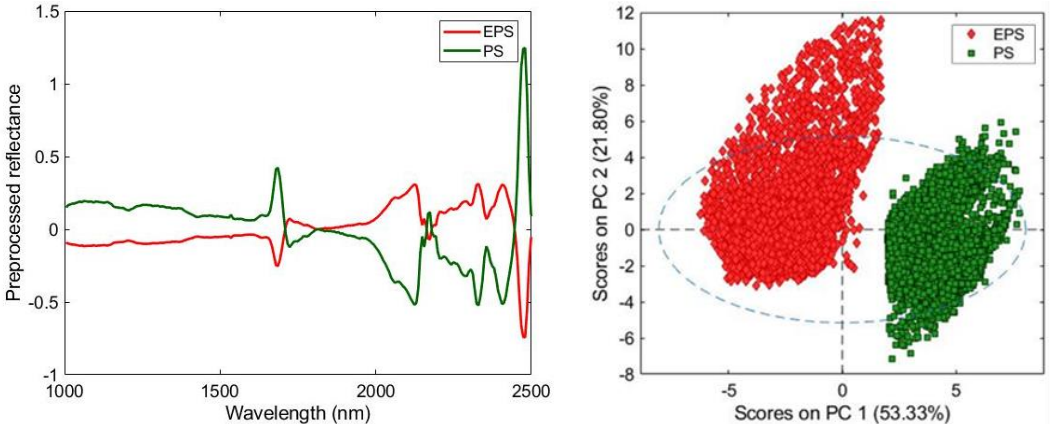

Figure S1. Preprocessed average spectra of polymers and corresponding PCA score plot related to the different rules used for the construction of the HI-PLS-DA model.

Table S1. *Sensitivity* and *Specificity* values in Calibration (Cal) and Cross Validation (CV) for each node of HI-PLS-DA model built for the recognition of microplastics.

**Classification rule**			**Class**		
**Node 0 Distinction between two classes: PA/PP/PE/PVC and PS/PET/EPS**			**PA/PP/PE/PVC**	**PS/PET/EPS**	
	Sensitivity	Calibration	0.998	1.000	
	Specificity		1.000	0.998	
	Sensitivity	Cross validation	0.998	1.000	
	Specificity		1.000	0.998	
**Node 1 Distinction between two classes: PA and PP/PE/PVC**			**PA**	**PP/PE/PVC**	
	Sensitivity	Calibration	1.000	1.000	
	Specificity		1.000	1.000	
	Sensitivity	Cross validation	1.000	1.000	
	Specificity		1.000	1.000	
**Node 2 Distinction between three classes: PE, PP and PVC**			**PE**	**PP**	**PVC**
	Sensitivity	Calibration	0.996	1.000	1.000
	Specificity		1.000	0.999	1.000
	Sensitivity	Cross validation	0.996	1.000	1.000
	Specificity		1.000	0.999	1.000
**Node 3 Distinction between two classes: PET and PS/EPS**			**PET**	**PS/EPS**	
	Sensitivity	Calibration	1.000	1.000	
	Specificity		1.000	1.000	
	Sensitivity	Cross validation	1.000	1.000	
	Specificity		1.000	1.000	
**Node 4 Distinction between two classes: PS and EPS**			**PS**	**EPS**	
	Sensitivity	Calibration	0.997	0.998	
	Specificity		0.998	0.997	
	Sensitivity	Cross validation	0.997	0.998	
	Specificity		0.998	0.997	

Table S2. Summary of the main shape and size parameters of the microplastic samples belonging to the different categories obtained by morphological and morphometrical analysis.

		Area (mm^2)	Aspect	Perimeter (mm)	Roundness	Feret min (mm)	Feret max (mm)	Feret mean (mm)
**Fragment**	**Min**	0.16	1.06	1.30	1.00	0.30	0.52	0.43
	**Max**	24.58	7.89	21.02	3.46	5.24	7.84	6.02
	**Mean**	3.16	1.89	6.46	1.45	1.35	2.42	1.96
	**St. Dev.**	4.68	0.92	4.51	0.41	0.95	1.67	1.32
**Filament**	**Min**	0.19	-	4.43	-	-	-	-
	**Max**	3.58	-	20.50	-	-	-	-
	**Mean**	1.07	-	10.00	-	-	-	-
	**St. Dev.**	1.10	-	6.25	-	-	-	-
**Granule**	**Min**	0.20	1.01	1.52	1.00	0.39	0.51	0.49
	**Max**	2.40	1.93	5.48	1.14	1.60	1.88	1.74
	**Mean**	0.67	1.16	2.72	1.01	0.79	0.94	0.87
	**St. Dev.**	0.47	0.17	0.87	0.03	0.27	0.29	0.27
**Foam**	**Min**	0.26	1.04	1.69	1.00	0.45	0.63	0.56
	**Max**	14.39	2.75	14.40	2.99	4.05	4.95	4.47
	**Mean**	2.44	1.56	5.85	1.33	1.40	2.07	1.77
	**St. Dev.**	2.28	0.49	2.64	0.35	0.67	0.92	0.77
**Pellet**	**Min**	0.36	1.02	2.00	1.00	0.60	0.71	0.65
	**Max**	6.51	1.32	9.30	1.10	2.60	3.38	2.93
	**Mean**	2.01	1.16	4.83	1.01	1.40	1.67	1.54
	**St. Dev.**	1.31	0.09	1.44	0.03	0.40	0.53	0.45

Area (mm^2): area of the object; *Aspect*: ratio between major axis and minor axis of ellipse equivalent to object. This parameter provides an indication of how much the particles are elongated; *Perimeter* (mm): length of the boundary of the object; *Roundness*: provides an indication of the circularity of the object (for a circle the parameter takes the value 1) and is calculated with the following equation: $(Perimeter)^2/4\pi Area$; *Minimum Feret Diameter* (mm): smallest caliper (Feret) length; *Maximum Feret Diameter* (mm): longest caliper (Feret) length; *Average Feret Diameter* (mm): average caliper (Feret) length.
